# Suppressing the Dielectric Loss in Superconducting Qubits through Useful Geometry Design

**DOI:** 10.3390/e24070952

**Published:** 2022-07-08

**Authors:** Haoran He, Weilong Wang, Fudong Liu, Benzheng Yuan, Zheng Shan

**Affiliations:** State Key Laboratory of Mathematical Engineering and Advanced Computing, Zhengzhou 450001, China; hehaoran0527@126.com (H.H.); wlwang19888@163.com (W.W.); lwfydy@126.com (F.L.); benzhengyuan@outlook.com (B.Y.)

**Keywords:** superconducting qubits, dielectric loss, energy participation ratio

## Abstract

Dielectric loss from different interfacial layers in the superconducting circuit and from external environment may cause superconducting qubit decoherence. Compared to modeling the entire device at once with a numerical solver, quantitatively formulating the dielectric loss can both describe all loss mechanisms and make the optimization more transparent. In this paper, we first analyze the expression formula of dielectric loss, and obtain a design scheme that can reduce the dielectric loss of qubits. That is, we replace the straight junction wires with the tapered junction wires. Based on this scheme, we perform a simulation to optimize the design of junction wires. Finally, a real experiment is conducted to verify our design. The results show that both the $T_{1}$ time and $T_{2}$ time of qubits are significantly improved.

## 1. Introduction

Superconducting quantum chips are currently one of the mainstream technologies for realizing quantum computers. Although the scale of qubits has reached a breakthrough to the order of one hundred [1], as the scale increases, the impact of dielectric loss becomes more and more prominent. Over the past decade, many optimization strategies to improve the quality of superconducting qubits have been proposed, including charge and flux noise suppression, detailed design of the electromagnetic environment in which superconducting qubits operate, the search for better materials and fabrication methods, and improved design to minimize equipment losses [2,3,4,5,6,7,8,9]. Dielectric loss, which is one of the earliest processes discovered to cause qubit relaxation, happens when energy is drawn from the qubit’s coherent state, making the qubit decay to the ground state from the excited state [10]. It has always been a key factor restricting the coherence time of superconducting qubits [11].

A superconducting qubit can be seen as a resonator composed of a capacitor and a nonlinear inductance in parallel, in which the superconducting Josephson junction provides the nonlinear inductance, and the “0” and “1” states of a qubit are formed by the two lowest energy levels. The structure connecting the Josephson junction to the parallel plate capacitor is called junction wire. The performance of superconducting quantum chips strongly rely on the circuit quality factor *Q*. For transmon qubits [12], the dielectric loss part of *Q* can be decomposed into contributions from different materials or regions [13]:

(1) $$Q^{-1}=\sum_{i}p_{i}\tan\delta_{i},$$

where tan $\delta_{i}$ denotes the loss tangent of interface type *i*, and $p_{i}$ is the energy participation ratio of interface type *i* (*i* = substrate–air (SA), metal–substrate (MS), and metal–air (MA)). To improve the factor *Q*, one can reduce dielectric losses by optimizing $p_{i}$ and tan $\delta_{i}$. The strong correlation between dielectric loss and tan $\delta_{i}$ will be studied later, and this paper mainly focuses on the influence of energy participation ratio on dielectric loss.

The participation ratio of various materials in the vicinity of the qubit, which represents the proportion of electric field energy contained within the material volume [14], is crucial in determining dielectric loss. It is given by:

(2) $$p_{i}=\frac{\epsilon_{i}/2}{W}\int dAt_{i}{\left|E_{i}\right|}^{2},$$

where $t_{i}$ is the thickness of the thin dielectric layer, dA is the surface integral instead of the normal volume integral, $E_{i}$ is the dielectric layer’s surface electric field, and $\epsilon_{i}$ is the dielectric layer’s dielectric constant.

The total energy of the capacitor is $W\equiv V^{2}C/2$, where *V* denotes the voltage and *C* denotes the total capacitance. Since the capacitance is easier to determine from the designed distance, it is straightforward to use the length *L* to characterize the qubit capacitance $C\equiv \epsilon_{0}L$ [15].

Three participation ratios can be considered for surface dielectrics [16]:

(3) $$p_{MA}=\frac{1}{\epsilon_{MA}}\frac{t_{MA}}{L\epsilon_{0}/2}\left[\frac{\epsilon_{0}}{2}\int_{MA}dA{\left|E_{0}/V\right|}^{2}\right],$$

(4) $$p_{MS}=\frac{\epsilon_{s}^{2}}{\varepsilon_{MS}}\frac{t_{MS}}{L\epsilon_{0}/2}\left[\frac{\epsilon_{0}}{2}\int_{MS}dA{\left|E_{0}/V\right|}^{2}\right],$$

(5) $$p_{SA}=\epsilon_{SA}\frac{t_{SA}}{L\epsilon_{0}/2}\left[\frac{\epsilon_{0}}{2}\int_{SA}dA{\left|E_{0}/V\right|}^{2}\right].$$

$\epsilon_{MS}$ is the dielectric constant of the MS interface layer; $\epsilon_{MA}$ is the dielectric constant of the MA interface layer; $\epsilon_{SA}$ is the dielectric constant of the SA interface layer. The formulas in parentheses represent the surface energy.

It can be clearly seen that generally, due to the large volume and dielectric constant of the substrate, the substrate is the region with the largest energy participation ratio in the qubit, and it is also the key region that determines the scale of the dielectric loss. However, previous studies have shown that the interfacial dielectric loss around the Josephson junction portion of the circuit is the main reason limiting the lifetime of the state-of-the-art transmon qubits. In this paper, the dielectric loss is reduced by optimizing the energy participation ratio $p_{i}$ through changing the device structure. In particular, the straight junction wires are replaced with the tapered junction wires.

To determine the surface participation ratios of three interfaces: SA, MS, and MA, the finite element method (FEM) technique has traditionally been employed to simulate the transmon qubit design [17,18]. Due to the huge disparity in thickness of these interfacial layers and the scale of the total qubit size or resonator length, precise determination of the surface participation ratios in these domains becomes problematic. Calculating the density of electric field on the interface surface is one answer to this problem. The participation ratio near the three critical interfaces in the plane design can then be computed analytically using conformal mapping based methods [5]. However, the premise of this approach is that the dielectric layer is thicker than the metal layer, which is contrary to the actual situation. The latest method is based on the method proposed in Ref. [6], making use of the corner field scaling and numerical simulation. This approach works with a lossy dielectric with a thickness of a few nanometers and a metal layer roughly 0.1 μm thick, which is similar to the realistic design of a superconducting quantum device.

**Figure 1:**
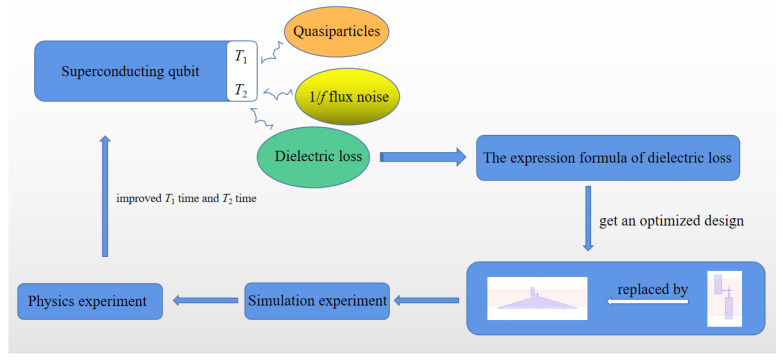
Overall framework. A qubit optimization design is obtained through the analysis of the dielectric loss formula. The design scheme is verified by performing simulation and physical experiments. It is verified that the $T_{1}$ time and $T_{2}$ time are both improved by our design.

In this paper, the energy participation ratio formulas of the junction wires are analyzed, and an optimized design that can reduce the dielectric loss is obtained. That is, the straight junction wires are replaced by the tapered junction wires. Then, the qubit structural model with tapered junction wires is established using HFSS software. By simulation, we obtain the electric field energy of the desiged chip. Based on the design parameters of superconducting qubits, we fabricate the chip and conduct a real experiment on the fabricated chip to verify our design. The overall framework of this paper is shown in Figure 1.

## 2. Methods and Fabrication

### 2.1. Comparison of Surface Energy between the Straight and Tapered Junction Wires

**Figure 2:**
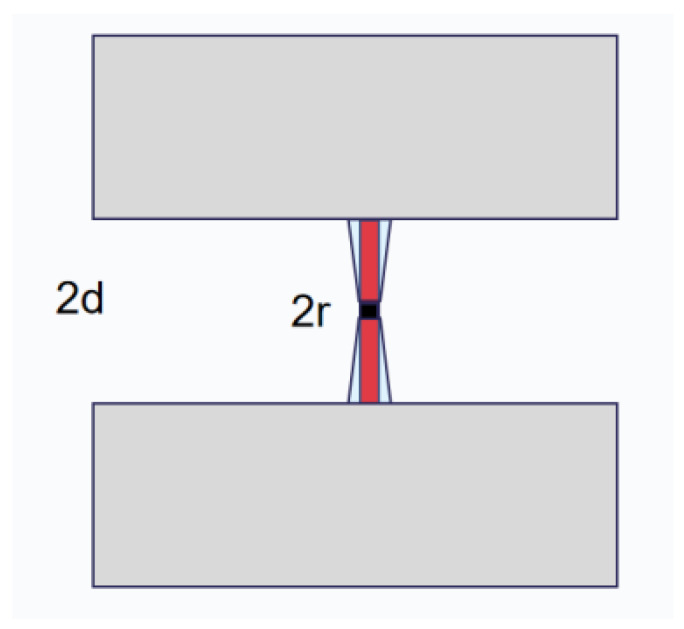
Diagram of the junction wires structure. The black square in the middle is the Josephson junction, the red wires are the straight junction wires, and the tapered junction wires are shown in light blue.

The Josephson junction and the capacitor electrodes are connected by two straight junction wires with a total length of 2*d* (Figure 2).

The metal surface energy of the straight wires of a constant width can be derived below [15]:

(6) $$U_{sw}^{m}\approx \frac{\epsilon V^{2}}{2}\frac{\ln\left(4r/t\right)+c_{m}}{\ln^{2}\left(d/r\right)}\frac{d}{r},$$

where $c_{m}$ is the angular correction for calculating the surface energy of the metal edge.

The surface energy of the substrate for the straight wires with the equal width is [15]:

(7) $$U_{sw}^{s}\approx \frac{\epsilon V^{2}}{4}\frac{\ln\left(4r/t\right)+c_{s}}{\ln^{2}\left(d/r\right)}\frac{d}{r},$$

where $c_{s}$ is the angular correction for calculating the edge surface energy of the substrate.

This surface energy can be large due to the *d/r* factor in Equations (6) and (7). Therefore, it could be better to taper the junction wires. Numerical integration of the wires energy gives the metallic surface energy of the tapered junction wires [15]:

(8) $$U_{tw}^{m}\approx 0.38\epsilon V^{2}\frac{\ln\left(d/r_{0}\right)}{S}\frac{\ln\left(4Sd/r\right)+c_{m}}{\ln^{2}\left(4/S\right)},$$

where *S* is the slope of the tapered junction wires. Note that, for simplicity but without loss of generality, in this paper only the case of *S* = 0.4 is considered. The substrate surface energy of the tapered junction wires are fitted as [15]:

(9) $$U_{tw}^{s}\approx 0.15\epsilon V^{2}\frac{\ln\left(d/r_{0}\right)}{S}\frac{\ln\left(4Sd/r\right)+c_{s}}{\ln^{2}\left(4/S\right)}.$$

**Figure 3:**
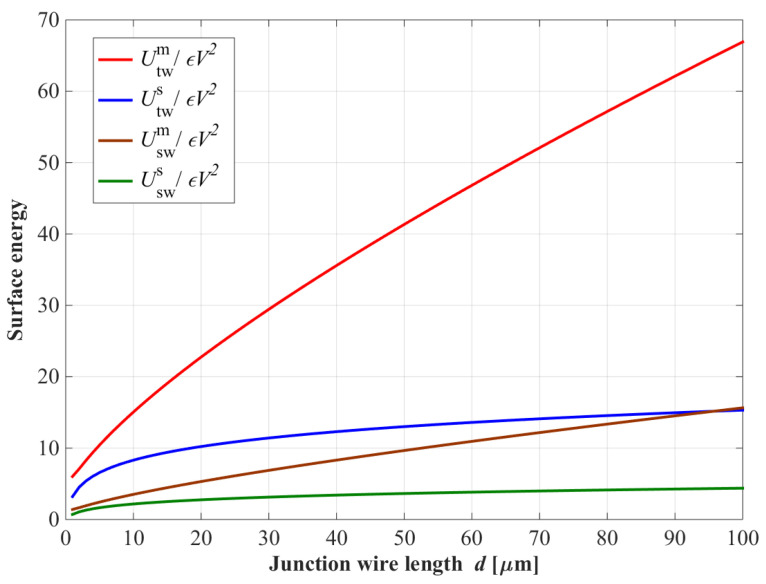
The relationship between the metallic surface energy of straight and tapered junction wires varies and length *d*. For wire lengths *d* ≥ 10 μm, the energy of the tapered junction wire is significantly reduced. The parameters are $t=r_{0}$ = 0.1 μm and *S* = 0.4.

The metallic and substrate surface energy for straight and tapered junctions are plotted in Figure 3. For small distances *d* ≤ 10 μm, the results of the two cases are similar. However, for *d* > 10 μm, the tapered loss decreases significantly. Therefore, optimizing the tapered junction wires design becomes increasingly important when using a larger value of *d*.

### 2.2. Design and Fabrication

**Figure 4:**
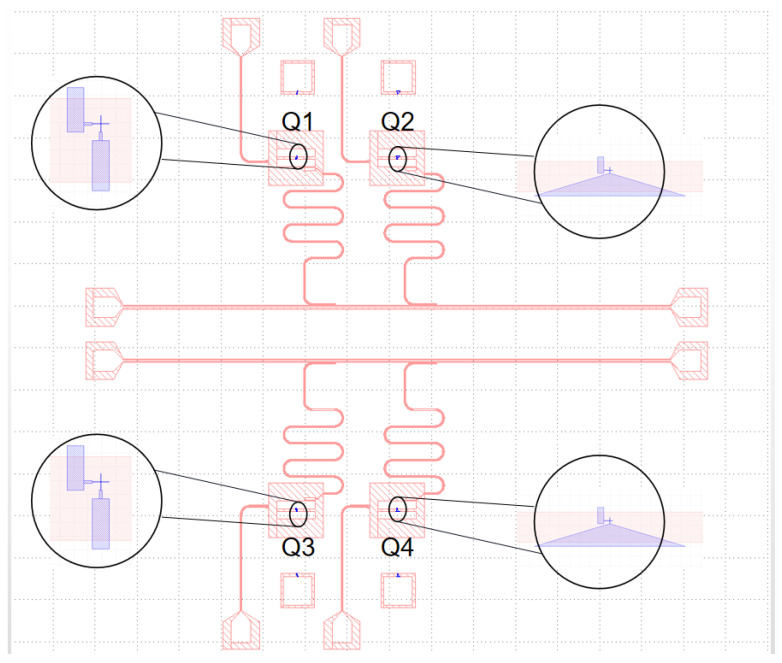
A planar-design 7.5 mm × 8 mm sample chip. The upper and lower transmission lines of the chip are of symmetrical. The structures of qubits Q1 and Q3 are the same, and both use the straight junction structure. The structures of qubits Q2 and Q4 are the same, and both use the tapered junction structure.

Since tan $\delta_{SM}$ is sensitive to the choice of materials and the fabrication process of the chip, we have fixed the choice of the substrate material of the chip, the material of the metal film and the fabrication process. In our design, the chip layout is symmetrical up and down as shown in Figure 4. Each part has two different junction wire structures. The spacing between the two qubits is 0.6 mm. The states of qubits Q1 and Q2 are readout via the the resonant cavities, whose frequencies are 6.1 GHz and 6.3 GHz, respectively. The chip fabrication process of the four qubits are the same. A 100 nm aluminum metal film is evaporated on a sapphire substrate by an electron beam evaporation process. We fabricated Manhattan-style [19,20] Josephson junctions using two aluminium leads, that extend from the pads and overlap each other, separated by an aluminium oxide barrier between them.

## 3. Results

### 3.1. Simulation Experiment

According to Equations (2)–(5), it can be concluded that the dielectric loss is proportional to the interface surface energy *U*. The value of *E* can be used to characterize the electric field energy, and thus the loss. Based on the designed superconducting chip structure and fabrication process parameters, the model of the superconducting qubit is established by HFSS. The model is divided into two layers. The upper layer is a two-dimensional qubit structure pattern, including: parallel plate capacitors, junction wires, and the coupling part between the qubit and the readout resonator. The metal material is aluminum and the substrate layer is sapphire with a dielectric constant of 10. Finally, the entire area is covered with a vacuum layer 4 times the thickness of the substrate. The Josephson junction is set as an inductor with a value of 15 nH.

**Figure 5:**
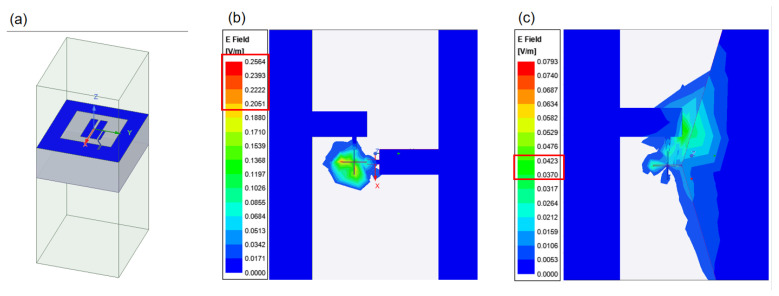
The simulated electric field energy at the substrate surface for two different junction wires. The value in the red box in the figure is the approximate electric field value obtained by the simulation. (**a**) The model of the superconducting qubit; (**b**) straight junction wires; and (**c**) tapered junction wires.

Dielectric loss affects the electric field around the qubit and causes energy relaxation. As shown in Figure 5, compared with the case of the straight junction wires (Figure 5b), the electric field distribution area on the surface of the Josephson junction is significantly reduced, the electric field intensity on the edge surface of the Josephson junction with tapered junction wires (Figure 5c) is significantly reduced as well. This means that by replacing the straight with the tapered wires, the dielectric loss can be reduced, and thus the qubit performance can be improved.

### 3.2. Physics Experiment

#### Experimental Setup and Wiring

**Figure 6:**
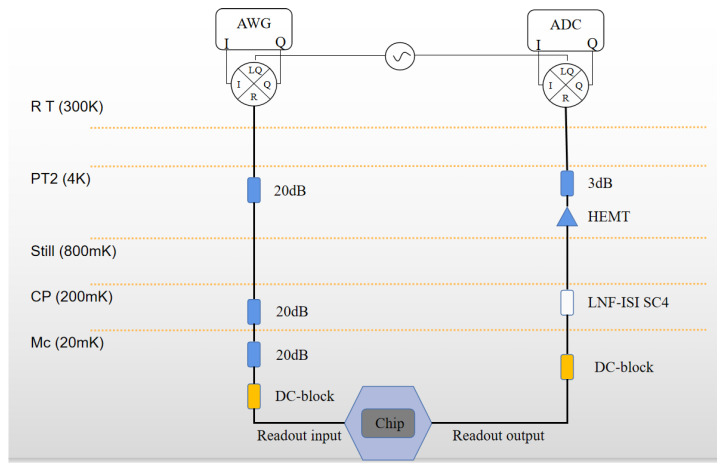
Low temperature test routing and signal synthesis at room temperature.

The chip sample is installed in a dilution refrigerator with a temperature of about 20 mK. The readout wiring scheme is shown in Figure 6. The test results are shown in Table 1.

From the test results shown in Table 1, the $T_{1}$ time and the $T_{2}$ time of the qubits with tapered junction wires are both higher than those of the qubits with straight junction wires. In specific, the average $T_{1}$ time has improved by about 60% and the average $T_{2}$ time has improved as high as 600%. Therefore, it is verified by the experiment that the design scheme with tapered junction wires can improve the performance of the superconducting qubits.

## 4. Conclusions

The formulas that can accurately calculate participation ratios of straight and tapered junction wires is introduced. By studying the formula to quantify the dielectric loss, a graphic design to reduce the dielectric loss is obtained. The traditional finite element method cannot be used to accurately calculate energy participation ratio due to the huge difference between the length and thickness of each structure of the qubit. In this paper, by analyzing the formula of the energy participation ratio, combined with the parameters of superconducting qubit design and process fabrication, the superconducting qubit model is established by HFSS simulation software. The electric field intensity obtained by the simulation indirectly indicates the energy participation ratio. The simulation results show that the energy participation ratio and the dielectric loss can be reduced by replacing the straight junction wires with the tapered junction wires. Finally, our characterization results of two groups of superconducting qubits with different junction wire structures show that the qubits with tapered junction wires have superior performance compared to the ones with straight junction wires. The results of this study provide a useful reference for the geometrical design of superconducting qubits. The next step is to study the influence of the slope *S* of the tapered junction wires on the dielectric loss to further improve the qubit quality.

## Figures and Tables

**Table 1:** Experimental data. The second column of data represents the shape of the junction wire: *S* is a straight type, and *T* is a tapered type. The third and forth columns represent the resonator frequency Fr and the qubit frequency Fq, respectively. The idea of measuring $T_{1}$ is to give the qubit a π-pulse at the initial moment to excite the qubit to the ∣1〉 state, and then measure changes of the qubit state over time. The measurement method of $T_{2}$ is the Ramsey measurement. At the initial moment, a π/2-pulse with a phase of 0 is given. After waiting for a certain interval time *t*, another phase is given. In the rotating reference frame, we can obtain a π/2-pulse at ∣1〉 probability, and finally use the fitting function to get the $T_{2}$ time of the system. The last two columns show the measured $T_{1}$ time and $T_{2}$ time.

Qubit	*S*/*T*	Fr (GHz)	Fq (GHz)	*T*_1_ (μs)	*T*_2_ (μs)
Q1	*S*	6.149	5.105	12.7	0.78
Q2	*T*	6.342	5.287	16.4	3.49
Q3	*S*	6.159	5.398	6.29	0.77
Q4	*T*	6.352	5.258	14.1	7.48

## Data Availability

The data that support the findings of this study are available from the corresponding author upon reasonable request.
